# Something New in Medicine

**Published:** 1915-10-09

**Authors:** 


### SOMETHING NEW IN MEDICINE.

The Preacher, as we know, has said there is nothing new, and has asked in incredulous tones for an experience of which it may be said, "See, this is new!" The generalisation is a large one, and though dignified by tradition and affirmed as a commonplace, is obviously incapable of proof. Of everything that has happened "under the sun" no man knoweth, or can know, and hence whether the wheel of history really moves forward, or, on the other hand, constantly returns on itself, is a debate as unprofitable as that which concerns the relative precedence of the hen and the egg. Suffice it that there are new experiences for the individual if not for the great time order, and it is with these we have to reckon our account.

Of no fashion of work can it be as truly said as of medicine that, however long the personal record, it may furnish at any moment something new. We are not referring to some startling discovery or important scientific development, but to the individual and personal experience of those engaged in medical practice. Arguing in advance, it might be anticipated that an experience of a moderate number of years would exhaust the possibilities of surprise, and that thereafter the practitioner would merely continue to see what he had seen before. Yet most certainly it is not so. To-morrow, perchance, any one of us, however senior his status, may meet in his work a symptom, a combination of symptoms, a clinical history or exhibition which he has never met before, and of which, equally, his colleagues must profess inexperience. The reasons for this are many, and prominent among them stands the something which we call "individuality," and which gives to each patient the particular qualities, or association of qualities, that distinguish him from his fellows and influence his reactions both to pathological and to therapeutic agencies. No clinical case is exactly the fellow of another, because no man is cast in the mould of his neighbour. There are those to whom this aspect of medicine has its gloomy side. They are oppressed by a sense of incompleteness, and long for the clean-cut rule which may be applied with certain and mechanical precision. Their ideal home is the completed and settled and well-ordered city, and it irks them to be of "such as dwell in tents." On the other hand are those who, in spite of its disturbing effects, rejoice in the free air of new experiences and the modifications of opinion which these impose. The "stagnation of your perfect state" has no attractions for these minds, nor the rigid formulas which spare the necessity for thought. For such, medicine is a discipline of life, and not achievement, but constant effort to achieve, the mental atmosphere to be desired. "To travel hopefully," as Robert Louis Stevenson puts it, "is a better thing than to arrive," though let us hope that both are possible.

It is the character of the practitioner's work here considered which rules, or ought to rule, his early training. If what has already been said is true, it is obvious that no curriculum can furnish the student with advance copies of all the experiences he is fated to meet. However "practical" his studies, something new will inevitably meet him on the morrow. What can be given to him, therefore, is not a map of the country he is about to cross—for this is known to no man—but a method of travelling. His equipment must be a certain mental attitude, a basis of fundamental facts, well-established principles, and fashions of working. It is these attributes which will fit him to meet the unknown problems and new situations that await him; and unless they are provided, he will be ill-supplied for his journey, even though he may boast practical experiences not a few. These experiences are, of course, valuable, and even necessary, but their full value only arrives when they are made occasions for the cultivation of the mental habits which secure the wise application of old truths to new circumstances. This training has its early, formal, and all-important period. But it has no ending, for the experience of to-day will not be repeated to-morrow, though the one properly used may yield the light by which the new event may be interpreted. It is an old art is medicine, but its practice has no record of monotony.

